# Detection of all four dengue serotypes in *Aedes aegypti* female mosquitoes collected in a rural area in Colombia

**DOI:** 10.1590/0074-02760150363

**Published:** 2016-04

**Authors:** Rosalía Pérez-Castro, Jaime E Castellanos, Víctor A Olano, María Inés Matiz, Juan F Jaramillo, Sandra L Vargas, Diana M Sarmiento, Thor Axel Stenström, Hans J Overgaard

**Affiliations:** 1 Universidad El Bosque, Grupo de Virología, Bogotá, Colombia; 2 Universidad El Bosque, Instituto de Salud y Ambiente, Bogotá, Colombia; 3 Durban University of Technology, South African Research Chair Initiative, Durban, South Africa; 4 Norwegian University of Life Sciences, Department of Mathematical and Technological Sciences, Ås, Norway; 5 Kasetsart University, Department of Entomology, Bangkok, Thailand; 6 Institut de Recherche pour le Développement, Maladies Infectieuses et Vecteurs Écologie, Génétique, Évolution et Contrôle, Montpellier, France

**Keywords:** dengue virus, RT-PCR, household, rural settlement, multiple infection, forecasting

## Abstract

The *Aedes aegypti* vector for dengue virus (DENV) has been reported in urban and periurban areas. The information about DENV circulation in mosquitoes in Colombian rural areas is limited, so we aimed to evaluate the presence of DENV in *Ae. aegypti* females caught in rural locations of two Colombian municipalities, Anapoima and La Mesa. Mosquitoes from 497 rural households in 44 different rural settlements were collected. Pools of about 20 *Ae. aegypti* females were processed for DENV serotype detection. DENV in mosquitoes was detected in 74% of the analysed settlements with a pool positivity rate of 62%. The estimated individual mosquito infection rate was 4.12% and the minimum infection rate was 33.3/1,000 mosquitoes. All four serotypes were detected; the most frequent being DENV-2 (50%) and DENV-1 (35%). Two-three serotypes were detected simultaneously in separate pools. This is the first report on the co-occurrence of natural DENV infection of mosquitoes in Colombian rural areas. The findings are important for understanding dengue transmission and planning control strategies. A potential latent virus reservoir in rural areas could spill over to urban areas during population movements. Detecting DENV in wild-caught adult mosquitoes should be included in the development of dengue epidemic forecasting models.

Dengue is currently regarded as the most important mosquito-borne viral disease globally (Bhatt et al. 2013, Murray et al. 2013). Dengue transmission has mainly been documented from urban areas, where it is transmitted by *Aedes aegypti* (L.) (San Martin et al. 2010). In Colombia, nearly 80% of the country has appropriate geographical and ecological conditions to allow mosquito breeding and dengue transmission (MinSalud 2012). The close contact between mosquitoes and humans results in high endemicity and frequent outbreaks (Ocampo & Wesson 2004). Up to the 1990’s dengue disease appeared mainly in adult populations, however since 2005, children under the age of 15 years have been the most affected group (Padilla et al. 2012). The disease seems to be in transition towards hyperendemicity, with all four dengue serotypes circulating in the country (Villar et al. 2015). During the last years, the number of dengue cases has increased in Colombia, including the department of Cundinamarca, where this study was carried out. In Cundinamarca, 444 dengue cases were documented in 2011, 785 in 2012, 2,291 in 2013, and 1,812 in 2014 (MinSalud 2013). The majority of cases occurred in urban areas.

Because of their domestic habits, *Ae. aegypti* is considered an urban mosquito, but it is also found in rural areas in Colombia (Morales 1981, INS 2012). More recently, our group found *Ae. aegypti* to be abundant in rural areas of the municipalities of La Mesa and Anapoima in the department of Cundinamarca (Olano et al. 2015). These two small towns are in the core area of a popular tourist region surrounded by large rural areas with intense commercial interchange characterised by high population movement. In addition, most of rural households do not have public services, such as piped water or sewage connections and have to store water in containers or drums, which are suitable breeding sites for *Ae. aegypti* (Romero-Vivas et al. 2006, Quintero et al. 2014).

Despite general information about the presence of *Ae. aegypti* in Colombia (Ocampo et al. 2011), little is known about their presence in rural areas. Similarly, virtually nothing is known about natural dengue virus (DENV) infection in mosquitoes in rural areas.

The objective of this study was, therefore, to evaluate the presence of the different DENV serotypes in mosquitoes collected in rural areas of La Mesa and Anapoima.

## MATERIALS AND METHODS

*Study area* - Anapoima and La Mesa municipalities are located about 60-90 km southwest of Bogotá, the capital of Colombia. Details of the study area are described in Olano et al. (2015). Briefly, Anapoima has a mean altitude of 710 m above sea level (asl), an annual rainfall of 1,300 mm, and an average temperature of 26ºC. La Mesa is located at an altitude of 1,200 m asl. The annual rainfall is 1,300 mm and the average annual temperature is 22ºC. In Anapoima, 57% of the approximately 12,000 inhabitants live in rural areas, while the corresponding figures for La Mesa is 45% of approximately 30,000 inhabitants. People in this region are mainly engaged in agriculture and tourism activities. The rural population generally live in settlements with dispersed houses, but also in so called *inspecciones*, which are more densely populated small village clusters, looking more like a small urban town*.* This study is the result of add-on investigations in relation to a larger a cluster randomised control study on dengue and diarrhoea and other research carried out in the study area (e.g., Overgaard et al. 2012, Olano et al. 2015). These municipalities were selected based on the presence of dengue in the region. The incidence of dengue in Anapoima was 377/100,000 inhabitants in 2012 and 619/100,000 inhabitants in 2013. The corresponding incidence for La Mesa was 230 and 627 per 100,000 inhabitants in 2012 and 2013, respectively (SIGIVILA/INS 2015). Universidad El Bosque (UEB) Ethical Committee approved the research protocol.

**Fig. 1:**
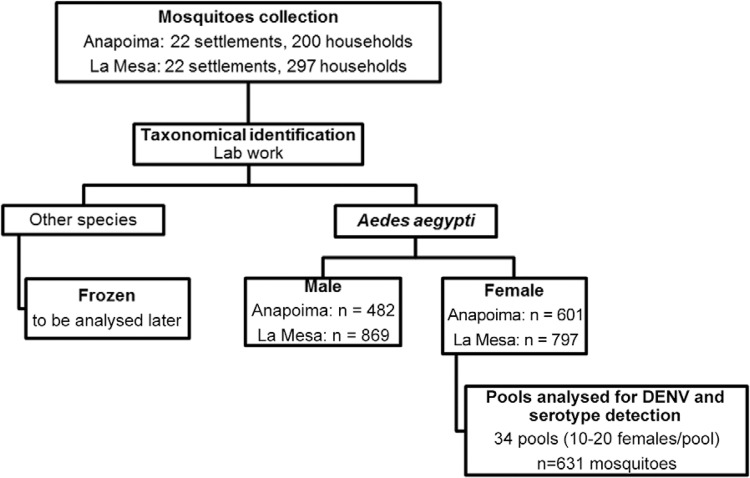
: flux diagram showing the sample processing. DENV: dengue virus.

*Mosquito collection* - Entomological surveys were carried out in rural households in La Mesa and Anapoima. Settlements were selected based on the presence of a public school, as we carried out a dengue and diarrhoea school-based project at the same time (Overgaard et al. 2012). Households were included if they were located near the school (< 200 m), if suspected dengue cases in that settlement were reported by the health municipal authorities, or if the inhabitants knew of suspected cases of dengue in the household or any nearby household during the last 30 days. Adult mosquitoes were collected indoors in 497 rural households in 44 different settlements, 22 in each municipality. Resting mosquitoes were captured at two occasions: July 2012 and April 2013, in each household using a Prokopack aspirator (Vazquez-Prokopec et al. 2009). Aspiration was done during 10 min in each household. Mosquito indoor resting density was calculated using data from each house. Mosquitoes were pooled by settlement and stored on dry ice before transportation to the Laboratory of Virology of the UEB, where they were stored at -80ºC until further processing. Mosquitoes were identified on a frozen table using morphological identification keys (Rueda 2004) and those identified as *Ae. aegypti* were separated by sex and counted. Generally, 20 female*Ae. aegypti* were pooled (1 sample) from settlements where sufficient mosquitoes were collected. Samples were stored in separate tubes for RNA extraction. Thus, 15 samples (pools) from 12 settlements in Anapoima and 19 samples from 15 settlements in La Mesa were analysed (Fig. 1, Table). Males were not processed. To avoid carry-over contamination during reverse transcription-polymerase chain reaction (RT-PCR), mosquito RNA isolation was performed in a separate room from that used to add enzymes and reagents for RT. PCR amplification and amplicon analysis were carried out in an independent room. Specific reagents, micropipettes, sterile tubes, and filter tips were used exclusively for each separate procedure following normal laboratory protocols.

**TABLE:** Number of *Aedes aegypti* mosquitoes collected from households in settlements in the municipalities of Anapoima and La Mesa, department of Cundinamarca, Colombia

Anapoima											
Settlement	Inspected households/ settlement (n)	*Ae. aegypti*			Female mosquito pool (n)		DENV serotype				
		
		Males (n)	Females (n)	Female density	Individual mosquitoes/ pool	Analysed	DENV positive	1	2	3	4
La Chica	11	15	17	1.55	17	1	1	X	X	-	-
Andalucia	10	9	3	0.30	-	-	-	-	-	-	-
Calichana	4	0	0	0	-	-	-	-	-	-	-
Golconda	7	2	1	0.14	-	-	-	-	-	-	-
La Esmeralda	10	6	1	0.10	-	-	-	-	-	-	-
La Esperanza	5	2	7	1.40	-	-	-	-	-	-	-
Panama	10	2	1	0.10	-	-	-	-	-	-	-
Inspeccion San Antonio^*a*^	24	112	157	6.54	60	3	1	-	X	-	-
El Rosario	9	9	8	0.89	-	-	-	-	-	-	-
La Guasima	5	5	5	1.00	-	-	-	-	-	-	-
Palmichera	2	0	0	0	-	-	-	-	-	-	-
Santa Ana	10	29	29	2.90	20	1	1	-	-	-	X
Lutaima	10	27	29	2.90	20	1	-	-	-	-	-
El Cabral	6	14	11	1.83	14	1	-	-	-	-	-
El Consuelo	10	20	10	1.00	10	1	-	-	-	-	-
El Copial	6	4	8	1.33	-	-	-	-	-	-	-
El Higueron	6	44	98	16.33	20	1	1	-	X	-	-
Las Mercedes	11	8	23	2.09	20	1	1	-	X	-	-
Santa Lucia	5	89	114	22.08	40	2	1	-	X	-	X
Patio Bonito	20	51	35	1.75	20	1	1	X	-	-	-
La Paz	17	9	13	0.76	13	1	1	-	X	-	-
San Judas	2	25	31	15.50	20	1	1	X	X	-	-
Total	200	482	601	3.01	271	15	9	3	7	0	2
La Mesa											
Settlement	Inspected households/ settlement (n)	*Ae. aegypti*			Female mosquito pool (n)		DENV serotype				
		Males (n)	Females (n)	Female density	Individual mosquitoes/ pool	Analysed	DENV positive	1	2	3	4
Anatoli	18	0	0	0	-	-	-	-	-	-	-
Payacal	18	9	10	0.56	10	1	1	X	-	-	-
Buenavista	7	19	21	3.00	20	1	1	-	X	-	-
Inspeccion San Joaquin^*a*^	50	155	148	2.96	60	3	2	X	X	-	X
La Concha	11	63	21	1.91	20	1	-	-	-	-	-
La Vega	10	13	4	0.40	-	-	-	-	-	-	-
Ojo de Agua	10	34	66	6.60	20	1	1	-	X	-	-
Capata	10	28	48	4.80	40	2	1	X	-	-	-
Inspeccion San Javier^*a*^	22	39	50	2.27	20	1	-	-	-	-	-
Alto del Frisol	10	0	0	0	-	-	-	-	-	-	-
Inspeccion La Esperanza^*a*^	26	15	9	0.35	-	-	-	-	-	-	-
Doima	12	0	0	0	-	-	-	-	-	-	-
Campo Santo	1	0	1	1.00	-	-	-	-	-	-	-
Florian	11	1	0	0	-	-	-	-	-	-	-
Alto de las Flores	10	30	22	2.20	20	1	1	X	X	-	-
Laguna Verde	10	46	43	4.30	20	1	1	-	X	-	-
Laguna Nueva	6	142	135	22.50	40	2	-	-	-	-	-
San Esteban	10	171	125	12.50	20	1	1	X	-	-	-
Santa Barbara	10	21	12	1.20	12	1	-	-	-	-	-
Guayabal	14	10	18	1.29	18	1	1	-	X	-	-
Zapata	14	20	36	2.57	20	1	1	-	-	X	-
Hungria	7	53	28	4.00	20	1	1	X	-	-	-
Total	297	869	797	2.68	360	19	12	6	6	1	1

*a*: *inspección* is a settlement with clustered houses in a confined area. More houses were sampled in these; DENV: dengue virus.

Individual mosquito infection rate was assessed by conservatively assuming that a pool positive for one serotype consisted of one single infected mosquito, a pool positive for two serotypes had two single infected mosquitoes etc. This assessment could have overlooked specimens with multiple infections and, thus, overestimated serotype infection rates. However, it is probably more likely that pools positive for two or more serotypes resulted from mosquitoes infected by a single serotype, rather than with multiple serotypes. This reasoning is also inherent in the concept of the minimum infection rate (MIR), which reflects the lower limit of the true infection rate assuming that only one infected individual exists in a positive pool, i.e., the chance of finding more than one infected individual in a positive pool is negligible (Gu et al. 2004). The MIR was calculated as the number of positive pools/total number of specimen assayed × 1,000.

*RNA extraction* -Wings and legs of female mosquitoes were removed. Mosquito thoraxes, abdomen, and heads in each pool were macerated in 300 ml of Dulbecco’s modified Eagle’s medium culture medium with antibiotics (penicillin-streptomycin) and foetal bovine serum. Samples were centrifuged and supernatants used to purify RNA using the QIAmp mini Viral RNA kit (Frentiu et al. 2014) and stored at -80ºC until further use. The RNA extraction process yielded adequate concentrations of RNA (between 312-1,135 mg/mL) to follow the amplification protocol.

*RT-PCR* - DENV were detected using 500 mg extracted RNA in a nested RT-PCR following the protocol described by Chien et al. (2006). Briefly, the first round was performed using mD1-TCAATATGCTGAAACGCGAGAAACCG and D2- TTGCACCAACAGTCAATGTCTTCAGGTTC that amplify a fragment of 511 base pairs. This PCR product was diluted 10-fold and amplified using mD1 primer and a primer set for the four dengue serotypes: rTS1-CCCGTAACACTTTGATCGCT (DENV-1), mTS2-CGCACAAGGGCATGAACAGTTT (DENV-2), TS3-TAACATCATCATGAGACAGAGC (DENV-3), and rTS4-TTCTCCCGTTCAGGATGTC (DENV-4). This yielded different amplicon sizes that were visualised on ethidium bromide-stained agarose gels. In the cases where DENV was detected, the second round was repeated using the primer pair specific to each serotype. Amplification products were separated by electrophoresis in 2% agar and evaluated against virus positive controls. The integrity of RNA was confirmed by amplifying a segment of mosquito mRNA to actin-1 using the primers and technique described in Staley et al. (2010). All samples successfully amplified the mosquito actin-1 gene. RNA isolated from supernatants of C6/36 mosquito cells infected with each DENV serotype were used as positive controls and supernatant of uninfected cells was used as a negative control. These RNA were processed independently and are the control batch routinely used in the laboratory.

## RESULTS

A total of 2,749 *Ae. aegypti* mosquitoes were identified (51% females, 49% males). The average mosquito density was 3.0 *Ae. aegypti* females/household in Anapoima and 2.7 in La Mesa (Table). However, there were large variations between settlements. The highest densities were found in Santa Lucia (Anapoima) and Laguna Nueva (La Mesa) with ca. 22 mosquitoes per household. There were two and four settlements with no *Ae. aegypti* mosquitoes in the samples in Anapoima and La Mesa (Table), respectively. Twelve settlements in La Mesa had more than 20 female *Ae. aegypti*of which three (Laguna Nueva, San Esteban, and San Joaquin) having more than 100 females. In eight settlements in La Mesa, less than 20 mosquitoes were collected, of which four had no *Ae. aegypti* females at all (Table).

**Fig. 2:**
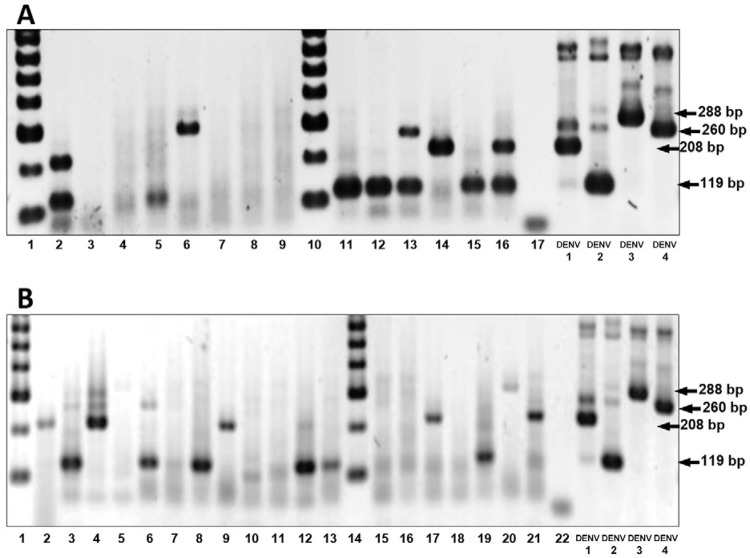
: agarose gels showing the amplification products generated from reverse transcription-polymerase chain reaction using RNA isolated from*Aedes aegypti* mosquito pools (from 10-20 mosquitoes in each) collected from settlements in Anapoima (panel A) and La Mesa (panel B). Detected dengue virus (DENV) serotypes are shown in parentheses. A: Anapoima. Lanes 1, 10: 100 bp marker; 2: La Chica (DENV-1 and DENV-2); 3: San Antonio 1 (Neg); 4: San Antonio 2 (Neg); 5: San Antonio 3 (DENV-2); 6: Santa Ana (DENV-4); 7: Lutaima (Neg); 8: El Cabral (Neg); 9: El Consuelo (Neg); 11: El Higuerón (DENV-2); 12: Las Mercedes (DENV-2); 13: Santa Lucia (DENV-2 and DENV-4); 14: Patio Bonito (DENV-1); 15: La Paz (DENV-2); 16: San Judas (DENV-1 and DENV-2); 17: Negative control; DV1-DV4: positive controls for DENV-1 to DENV-4; B: La Mesa. 1, 14: 100 bp marker; 2: Payacal (DENV-1); 3: Buenavista (DENV-2); 4: San Joaquín 1 (DENV-1); 5: San Joaquín 2 (Neg); 6: San Joaquín 3 (DENV-2 and DENV-4); 7: La Concha (Neg); 8: Ojo de Agua (DENV-2); 9: Cápata 1 (DENV-1); 10: Cápata 2 (Neg); 11: San Javier (Neg); 12: Alto de las Flores (DENV-1 and DENV-2); 13: Laguna Verde (DENV-2); 15: Laguna Nueva 1 (Neg.); 16: Laguna Nueva 2 (Neg); 17: San Esteban (DENV-1); 18: Santa Bárbara (Neg); 19: Guayabal (DENV-2); 20: Zapata (DENV-3); 21: Hungría (DENV-1); 22: Negative control; DV1-DV4: positive controls for DENV-1 to DENV-4; Neg: no DENV detected.

**Fig. 3:**
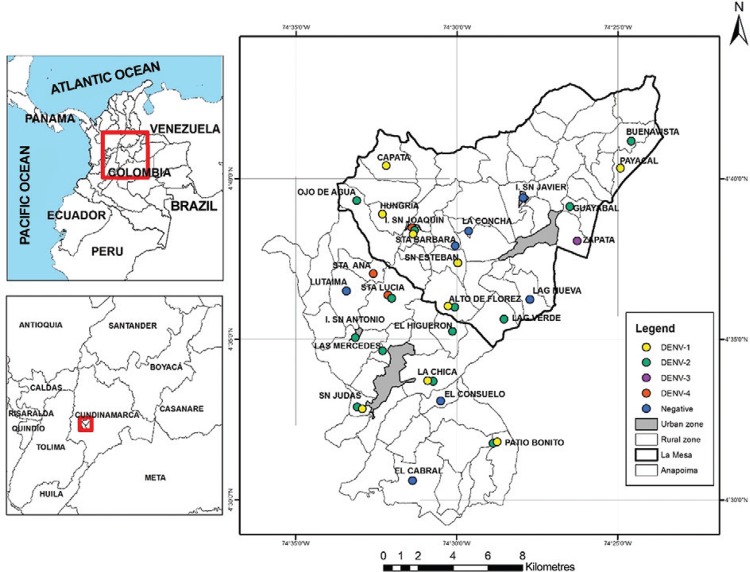
: map of the municipalities of Anapoima and La Mesa, department of Cundinamarca, Colombia, showing rural localities where dengue virus (DENV) serotypes were detected in *Aedes aegypti* female mosquito pools. Mosquitoes collected in July 2012 and April 2013.

Overall, 20 of the 27 (74%) settlements from which mosquitoes were analysed were positive for DENV. The sample positivity rate was 61.8% (21/34). In Anapoima, nine out of 12 analysed settlements (75%) were positive for DENV with DENV-2 serotype being the most frequent (7 of 12 settlements), followed by DENV-1 (3 of 12) and DENV-4 (2 of 12). Both DENV-1 and DENV-2 were found simultaneously in each of La Chica and San Judas settlements in Anapoima. In Santa Lucia settlement, both DENV-2 and DENV-4 were detected simultaneously in the same pool. There was no DENV-3 found in Anapoima (Fig. 2). In La Mesa, 11 out of 15 settlements (73%) were positive for DENV with DENV-1 and DENV-2 serotypes being most abundant found in six of the 15 analysed settlements. DENV-3 and DENV-4 were found in one settlement each. DENV-1 and DENV-2 were detected simultaneously in a single mosquito pool from Alto de las Flores settlement. Co-detection of DENV-2 and DENV-4 was made in a single pool from San Joaquin. DENV-3 and DENV-4 were simultaneously found in samples from Zapata and San Joaquin settlements (Table). The settlements where DENV positive mosquitoes were detected are summarised in Fig. 3. The proportion of DENV positive individual mosquitoes was assessed to 4.12% (26/631 × 100), corresponding to 26 assumed infected mosquitoes (12 detected serotypes in Anapoima and 14 in La Mesa) (see criteria in Materials and Methods and Table) in a total of 631 mosquitoes processed across all processed pools. The MIR was 33.3 (21 positive pools/631 total processed individual mosquitoes × 1,000). It is possible that several mosquitoes per pool were infected with the same serotype, thereby increasing the proportion of infected individual mosquitoes. However, as the study design did not allow these to be distinguished from each other, we decided to estimate the proportion as conservatively as possible.

## DISCUSSION

This study showed that *Ae. aegypti* is abundant in rural areas in Colombia with all four DENV serotypes present at high frequencies within a small study area. DENV infected mosquitoes were detected in 60% of the mosquito pools and 74% of the household settlements where mosquitoes were collected.

We found relatively high mosquito densities in the study area with an average of up to three *Ae. aegypti* per household (10 min electric aspirating per house). A quantitative comparison with other studies is difficult, due to different collection methods. However, studies in Thailand and Puerto Rico were performed in a similar way (10-min aspirating collections) with reported mean densities of 11 and seven *Ae. aegypti* per house, respectively (Scott et al. 2000). In Mérida, Mexico, a mean indoor abundance of close to eight *Ae. aegypti* females per house was found during the peak month of August (using a CDC backpack aspirator for 20 min) (Garcia-Rejon et al. 2008). We decided to pool mosquitoes from settlement for two reasons. Firstly, a single house frequently had less than three female mosquitoes, and secondly, the settlement behave as a community where people share and move permanently inside the settlement despite that houses are separated.

All four DENV serotypes have been in circulation in Colombia annually since 2006, based on serum samples taken from febrile patients (INS 2011). DENV-1 and DENV-2 were the most common serotypes found (80-90% of serum samples taken in 1975-2005). DENV-3 first appeared in 2005 and represents approximately 34.5% of detected serotypes in the country in recent years (Padilla et al. 2012). Compared with these frequencies we found 35% DENV-1, 50% DENV-2, 4% DENV-3, and 11% DENV-4 in our samples, indicating a large variation in temporal and spatial distribution of serotypes.

In Colombia, DENV has been detected in *Ae. aegypti* in urban areas (Groot et al. 1996, Romero-Vivas et al. 1998, Méndez et al. 2006), but no studies, so far, have investigated DENV infection rates in mosquitoes in rural areas. We found that 21 out of 34 pools (62%) contained mosquitoes infected with DENV. This is high compared to Groot et al. (1996), who detected DENV in 33% (9/27) of their pools collected in Armero (Colombia) or Guedes et al. (2010), who found 11% (9/83) of adult mosquito pools and 12% (17/139) of egg pools DENV positive in Brazil. The reason for the high rates of infection in our study could have been exacerbated by the Colombian dengue epidemic in 2013. The exact mosquito infection rate is not known since pools of mosquitoes were analysed. However, the estimated mosquito infection rate was calculated at 4.12% and the MIR was 33.3/1,000 mosquitoes. Studies carried out in the department of Córdoba (Colombia) in 1972 found an 1.9% DENV infection rate in*Ae. aegypti*, using similar calculations (Groot et al. 1996). During dengue epidemics in the 1970s, infection rates detected by immunofluorescence of individually tested mosquitoes collected from several Colombian urban locations were 2.9% (1/34), 3.6% (6/167), 5.5% (20/360), 6.7% (4/61), and 7.4% (11/149) (Groot et al. 1996). During a nonepidemic period in the department of Antioquia, a mosquito infection rate of 1.2% (24/2,065) in individual mosquitoes was found (Romero-Vivas et al. 1998). In southern Thailand, infection rates of 5-26% in individually tested mosquitoes were found in different locations (Thavara et al. 2006) and in Mexico and Brazil the urban *Ae. aegypti* infection rates were also high (Costa et al. 2009, de la Mora-Covarrubias et al. 2010). The mosquito infection rates in our study can be considered relatively high compared to other studies performed in urban neighbourhoods. It is clear that the high percentage of positive pools was not because of carry-over contamination, because different pools simultaneously processed were positive to different virus (amplicons) or were definitely negative.

Dengue control activities are not carried out in rural areas in Colombia. Almost 80% of rural households in Colombia lack basic water and sanitation facilities, such as piped water, sewerage, and solid waste collection (DANE 2013). The necessity to store water in households and the presence of solid waste therefore create abundant breeding sites for *Ae. aegypti* (Cabezas et al. 2011,Olano et al. 2015). As most dengue infections in humans are asymptomatic (Méndez et al. 2006, JE Castellanos et al., unpublished observations), the circulation of the virus in the mosquito population and among human hosts could be maintained for long periods in rural areas. The study area is characterised by high human population movements between urban and rural areas. The high infection rates in mosquitoes found here and the factors mentioned above suggest a latent virus reservoir in rural areas, which can spill over into urban areas at times of high population movements potentially creating epidemics.

Co-infection of different DENV serotypes in individual mosquitoes is not common. It was first reported from mosquitoes in southern Thailand (Thavara et al. 2006). We detected double DENV serotypes in four mosquito pools from single households and triple presence in one pool. It is more likely that these reflect single infections in separate mosquitoes and not multiple DENV detection in individual mosquitoes. Simultaneous transmission of several DENV serotypes in a small area could cause future severe dengue related with sequential secondary infection in the same individual. Experimentally, larvae can be infected with up to three DENV serotypes (Bara et al. 2013) and despite the lack of reports of natural infections with four serotypes in mosquitoes or humans, that scenario could theoretically be possible in intense transmission areas like in Colombian hyperendemic zones. We did not analyse infectivity in male mosquitoes in this study. However, males can also be infected with DENV (Thavara et al. 2006) and could play a role in virus transmission by vertically transmitting virus to offspring and females (Bara et al. 2013), and act as a reservoir of viruses during interepidemic periods which could favour their re-emergence in isolated areas.

Continuous dengue surveillance is important for detecting and predicting outbreaks, understanding spatial and temporal disease dynamics and evaluating control interventions (WHO 2009). Dengue epidemiological surveillance in Colombia is mainly based on case reports from health centres with dengue cases confirmed by serological but not virological diagnosis. A well-functioning early warning system for impending dengue epidemics does not exist to date. Traditional *Stegomyia* indices have not proved to be robust indications of dengue transmission (Bowman et al. 2014). A correlation between mosquito infection and dengue case number has been found in Colombia (Groot 1980, Méndez et al. 2006). Therefore, detection of DENV in wild mosquitoes could be used to develop new indicators that are more sensitive to forecast epidemics than the current ones. Further research is needed to find a robust forecasting model for endemic areas that is sensitive to local conditions.
